# New Anodic Discoloration Materials Applying Energy-Storage Electrochromic Device

**DOI:** 10.3390/ma16155412

**Published:** 2023-08-02

**Authors:** Po-Wen Chen, Chen-Te Chang

**Affiliations:** Division of Physics, Institute of Nuclear Energy Research, Taoyuan City 325207, Taiwan

**Keywords:** iridium oxide (IrO_2_), energy-storage electrochromic device (ESECD), cathodic arc plasma (CAP)

## Abstract

We have assessed new anodic coloring materials that can be used as ion storage layers in complementary energy storage electrochromic devices (ESECDs) to enhance their electrochromic storage performance. In our study, we fabricated counter electrodes (ion storage layers) using an IrO_2_-doping NiO (Ir:NiO) film through cathodic arc plasma (CAP) with varying surface charge capacities. We have also investigated the influence of a MoO_3_-doped WO_3_ (Mo:WO_3_) film using various Ar/O_2_ gas flow ratios (1/4, 1/5, and 1/6, respectively). The ESECDs used in the demonstration were 10 × 10 cm^2^ in size and achieved an optical transmittance modulation of the Ir:NiO ESECDs (glass/ITO/ Mo:WO_3_/gel polymer electrolytes/ Ir:NiO/ITO/glass), with ΔT = 53.3% (from T_bleaching_ (66.6%) to T_coloration_ (13.1%)). The ESECDs had a quick coloration time of 3.58 s, a rapid bleaching time of 1.24 s, and a high cycling durability. Furthermore, it remained at a 45% transmittance modulation level even after 3000 cycles. New anodic materials can thereby provide an alternative to traditional active materials for bi-functional electrochromic batteries.

## 1. Introduction

With the accelerating depletion of fossil fuels accompanied with increasing environmental degradation, energy conservation and emission reduction have become an inevitable global trend [1,2]. Currently, over 30% of the world’s energy consumption is dedicated to providing heating, cooling, ventilation, and artificial lighting for buildings [3,4,5]. One of the primary factors that affect the energy consumption of buildings is the windows, as a significant percentage of light and heat is transmitted and lost through them [6]. Electrochromic (EC) materials can reversibly adjust their optical characteristics, including reflectance, transmittance, and absorption, to regulate indoor sunlight and solar heat. EC materials can effectively reduce the heating and cooling loads inside a building [7]. Smart windows made of electrochromic materials can be utilized in architectural buildings’ rooms (Sage Glass, View, Inc., USA), auto-dimming rearview mirrors, and aircraft (Gentex Corp., USA) [8].

Energy storage electrochromic devices (ESECDs) consist of anodic and cathodic coloring materials arranged in a five-layer structure. This structure includes a pair of transparent conducting layers, an ionic conduction layer (electrolyte) in contact with an electrochromic (EC) layer, and a complementary ion storage layer [9,10,11,12]. Smart windows are made of electrochromic materials that can block solar and indoor sunlight heat, resulting in a reduction in the level of air-conditioning energy consumption [12,13,14,15,16]. The active electrochromic materials used in the device have undergone extensive assessments. Several transition metal oxides, such as WO_3_, V_2_O_5_, NiO, and TiO_2_, have been utilized in the form of supercapacitors or electrochromic electrodes, which exhibit good electrochromic properties, and their capacitive processes are typically accompanied by obvious yet tender color changes [17,18,19,20]. Electrochromic devices (ECDs) can function at low voltages and change color or bleach through the injection or extraction of positive ions (such as lithium or protons) and electrons into and out of the electrochromic materials [19,20,21].

Implementing the combination of these bi-functional electrochromic and energy storage properties through reversible redox reactions using an active electrode material has been a logical focus for many investigations [22,23,24,25]. It would be exciting to not only utilize smart windows, but also charge capacity, which possess excellent electrochromic and energy-storage capabilities as well as displaying significant color variations [22]. The general objective is to develop energy-saving strategies to overcome challenges in response to the worldwide energy crisis. In this context, energy-storage electrochromic devices (ESECD) are considered promising research possibilities due to their low power consumption, reversible color changing, low power driving, extensive optical modulation, and good memory characteristics [23,24,25,26].

Due to their potential use in heat-insulating glass for airplanes and smart windows for buildings, these devices are attracting a lot of attention [27,28]. WO_3_ is a widely recognized cathode material, while NiO is a frequently used anode material. The primary drawbacks of the NiO-ECD are its low contrast in optical transmittance and limited lifespan [29,30]. The IrO_2_ film has been proposed as an anodic electrode in ECDs, as it facilitates reversible oxidation and reduction reactions, enabling Li ions to enter and exit the interface between the electrodes and the electrolyte [29,31,32]. In general, electrode films for ECDs consist of an anode and a cathode that can be manufactured using various methods, such as sputtering [33,34,35], chemical deposition [36,37], sol-gel [38,39], dip-coating [40,41], pulsed-laser deposition [42], and electrodeposition [43]. In previous work by the authors, they already fabricated anodic coloring materials (IrO_2_ and NiO) and cathodic coloring materials (WO_3_) using a CAP process. The CAP technique is not widely applied due to its poor macro-particle production. Their inferior performance is caused by the consequence of plasma-liquid pooling on cathode spots and its attachment to the electrode film. These harmful macro-particles are the main factor underlying why the CAP technique is not suitable for industrial applications. Therefore, they have amended a way that made use of the Thornton deposition [19,20,21,31,32] to reduce the macro-particle size and adjust the process parameters for high work pressure to improve the quality.

**Figure 1:**
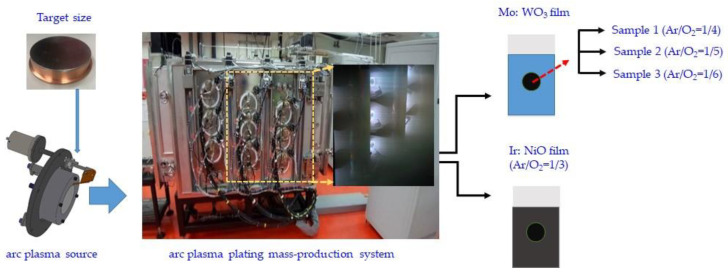
Schematic representation for the CAP-based technique synthesis of Mo:WO_3_/ITO under various Ar/O_2_ gas flow ratios (1/4, 1/5, and 1/6, respectively) and Ir:NiO Ar/O_2_ gas flow ratios (1/3) films.

In our study, we have examined the influence of the Mo:WO_3_/ITO film on the electrochemical and optical characteristics, including surface diffusion coefficients and optical density, under various Ar/O_2_ gas flow ratios (1/4, 1/5, and 1/6, respectively). The configuration of Ir:NiO ESECDs (glass/ITO/Mo: WO_3_/gel polymer electrolytes/Ir:NiO/ITO/glass) is illustrated in Figure 1. In addition, the ESECD measures the time it takes for coloring and bleaching to occur, as well as its cycling durability.

## 2. Materials and Methods

### 2.1. Synthesis of Mo:WO_3_ (Electrochromic Layer) Working and the Transparent Electrode

The electrochromic Mo:WO_3_ (MoO_3_-doped WO_3_ films) electrode was fabricated with the CAP technique using a molybdenum (Mo)–tungsten (W) alloy metal target (characterized with a 99.99% purity); the Mo/W weight ratio of the target was around 25%, while the deposition temperature was fixed at 50 °C. In the CAP technique, a Mo/W-metal target disk of 3 in was used. The target size consisted of a diameter of 3 mm and a thickness of 3 mm. The base chamber pressure was set to less than 6 × 10*^−^*^6^ Torr using a turbo pump. Mo:WO_3_ films (samples 1–3) in a series of reaction Ar/O_2_ gas flow ratios (1/4, 1/5, and 1/6, respectively) as electrochromic electrodes were deposited on indium tin oxide (ITO) glass. During this process, each ITO-coated glass sample was deionized for 2 min to remove surface-bounded particles. Indium tin oxide (ITO, Solaronix SA, Aubonne, Switzerland) with a 5.8 sheet resistance-coated glass was cut into wafers (10 *×* 10 cm^2^) for use as a transparent conducting substrate in the ESECDs. The deposition parameters are detailed in Table 1. The argon insert flow and oxygen reactive flow were controlled individually via mass flow controllers. In this work, we considered three different oxygen mass flows of 600, 750, and 900, with a fixed 150 sccm argon mass flow, along with their corresponding working pressures of 1 *×* 10^−2^ torr, 1.5 *×* 10^−2^ torr, and 3 *×* 10^−2^ torr, respectively. The 300 nm thickness ITO-coated glass with a high visible light transparency of 82% was used. The 200 nm thickness Mo:WO_3_ film was deposited using CAP technology as a working electrode (Mo:WO_3_ film on ITO/glass).

### 2.2. Deposition of IrO_2_ and the NiO Counter Electrode

The Ir:NiO layer was fabricated using the CAP technique, which utilized metallic iridium (Ir)–nickel (Ni) alloy metal targets (99.99% purity) with an Ir/Ni weight ratio of approximately 20%. The counter electrode was deposited on a 10 × 10 cm^2^ indium tin oxide (ITO) glass substrate using a fixed Ar/O_2_ gas flow ratio of 1/3. The deposition parameters implemented are detailed in Table 2. In this work, we considered a fixed 150 sccm argon mass flow along with 450 sccm oxygen mass flows, and the corresponding working pressure was measured as 8 *×* 10^−3^ Torr. The 300 nm thickness ITO-coated glass with a high visible light transparency of 82% was used. The 100 nm thickness Ir:NiO film was deposited using CAP technology as a counter electrode (Ir:NiO film on ITO/glass).

### 2.3. Gel Polymer Electrolyte Preparation

The electrolyte system consisted of a 10 wt% solution of 89–98 k PVA (99% hydrolyzed) in a solvent mixture of 80:20 dimethyl sulfoxide (DMSO):H_2_O. The PVA was first dissolved by stirring at 90 °C for 2 h and then subjected to two freeze–thaw cycles under a vacuum to produce PVA gels. Following the freeze–thaw process, the PVA gels were immersed in three separate fresh DI water baths for 1 h and were subsequently soaked in DI water for an additional 24 h to remove excess DMSO and form PVA hydrogels. These hydrogels were then immersed in baths containing BiCuClO_4_ with a 0.1 wt% PVA additive electrolyte for 24 h, allowing the water in the hydrogel to be replaced with sufficient liquid electrolyte, resulting in the formation of the PVA gel polymer electrolyte (GPE) [44].

### 2.4. Experimental Details

The process of ESECD fabrication involved respectively depositing a Mo:WO_3_ film/ITO on a glass substrate along with a counter film (Ir:NiO film on ITO/glass), and then fitting the two components together and sealing them using an epoxy adhesive. It is important to note that glass beads were used as spacers to maintain a cavity between the EC film and the counter film to hold the gel polymer electrolyte. Note that a small gap was also created in the epoxy for their use as an inlet into the space, which was sandwiched between two transparent conducting layers, which were in turn sandwiched between the two glass substrates. Dispensing for one side through pre-gluing with a gel polymer electrolyte ion injection in a vacuum pump were set as the production components. Scanning electrochemical characteristics were analyzed with the cycle voltammetry (CV) and chronoamperometry (CA) techniques using an Autolab PGSTAT30 model (Utrecht, The Netherlands) in a three-electrode system. The working electrode consisted of Ir:NiO/ITO/glass, the counter electrode was a platinum mesh, and the reference electrode was Ag/AgCl. The optical transmittance of the film was measured using an ultraviolet–visible (UV–Vis) spectrophotometer (model DH-2000-BAL, Ocean Optics, Dunedin, FL, USA) in the wavelength range from 300 nm to 900 nm, respectively, while in their coloration and bleached states.

## 3. Results

### 3.1. Mo:WO_3_/ITO Films: Electrochromic and Capacitive Performance

**Figure 2:**
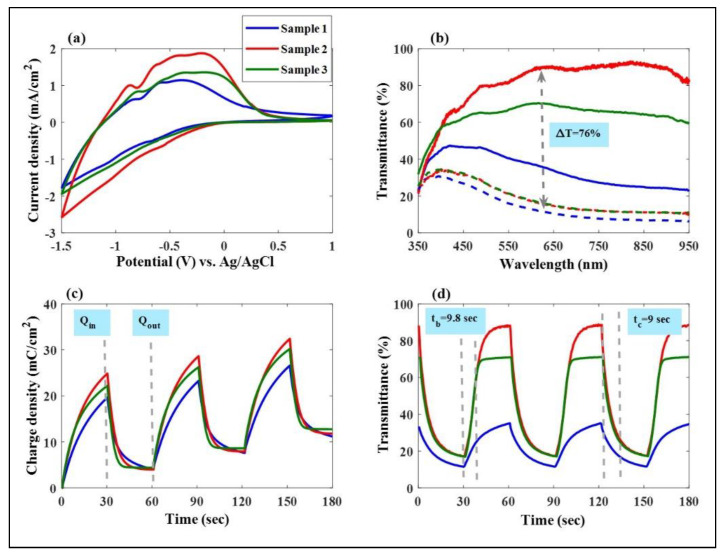
(**a**) Cycle voltammetry (CV) curves of Mo:WO_3_ films produced with different Ar/O_2_ gas flow ratios (1/4, 1/5, and 1/6, respectively) on ITO glass as a working electrode in a 0.5 M LiClO_4_/Perchlorate (LiClO_4_/PC) solution, a counter electrode (Pt mesh), and a reference electrode (Ag/AgCl). (**b**) Optical transmittance of the Mo:WO_3_ films with different Ar/O_2_ gas flow ratios for the coloring/bleaching states. (**c**) Surface charge capacities of the Mo:WO_3_ layers were determined using the intercalation surface charges (Q_in_) and extraction surface charges (Q_out_). (**d**) The in situ optical transmittance of the Mo:WO_3_ films at a wavelength of 633 nm.

We have investigated the electrochemical and energy storage properties of Mo:WO_3_/ITO/glass by constructing three-electrode cells. These cells consisted of a working electrode (Mo:WO_3_ film on ITO/glass), a counter electrode (Pt mesh), and a reference electrode (Ag/AgCl) in a 0.5 M LiClO_4_/Perchlorate (LiClO_4_/PC) solution. Figure 2a displays the cycle voltammetry (CV) curves of these Mo:WO_3_ films produced with different Ar/O_2_ gas flow ratios (1/4, 1/5, and 1/6, respectively) on ITO glass. The samples are denoted as Sample 1 (blue line), Sample 2 (red line), and Sample 3 (green line). The reaction that pertains to the colored and bleached states is described by Equation (1):

(W_1–y_Mo_y_)O_3_ (bleaching) *+* x(Li^+^ + e^−^) ⇔ Li_x_(W_1–y_Mo_y_)O_3_ (coloration). (1)

In order to achieve an optimal performance of the Mo-doped WO_3_ films deposited on ITO glass, cyclic voltammetry (CV) curves were conducted by scanning the potential from *−*1.5 V (coloring) to 1 V (bleaching) at a fixed rate of 0.1 V/s for the first cycle, respectively. Sample 2 exhibited a larger envelope area and a much higher peak current compared to Samples 1 and 3, respectively, indicating a greater participation of the Li^+^ charge in the electrochemical redox reaction [45,46,47,48].

Figure 2b illustrates the optical transmittance of these Mo:WO_3_ films at a wavelength of 633 nm under the same voltage range from *−*1.5 V to 1 V, respectively, with different Ar/O_2_ gas flow ratios indicating the coloring/bleaching effect. As shown in Figure 2b, Sample 2 displayed an extremely high transmittance modulation of 76% (89% in the bleached state and 13% in the colored state, respectively) at 633 nm, which is significantly greater than that of Sample 1 (27% modulation, with 39% in the bleached state and 12% in the colored state, respectively) and Sample 3 (58% modulation, with 71% in the bleached state and 13% in the colored state, respectively).

Figure 2c shows that the surface charge capacity of these Mo:WO_3_ layers was determined by integrating the CA curves and ranged from *−*1.5 to 1 V, respectively, versus AgCl/Ag for the intercalation surface charges (Q_in_) and extraction surface charges (Q_out_). It is clearly observed in Figure 2c that the Mo:WO_3_ films with the different Ar/O_2_ gas flow ratios of 1/4, 1/5, and 1/6 exhibited slight intercalation surface charges (Q_in_) of 19.65, 24.84, and 22.19 mC/cm^2^ and extraction surface charges (Q_out_) of 15.63, 20.85, 17.8 mC/cm^2^, respectively. The speed at which the electrochromic system switches from one state to another is a crucial factor in its practical application. This can be investigated through chronoamperometry, and the corresponding in situ transmittance at 633 nm has been depicted in Figure 2d.

**Figure 3:**
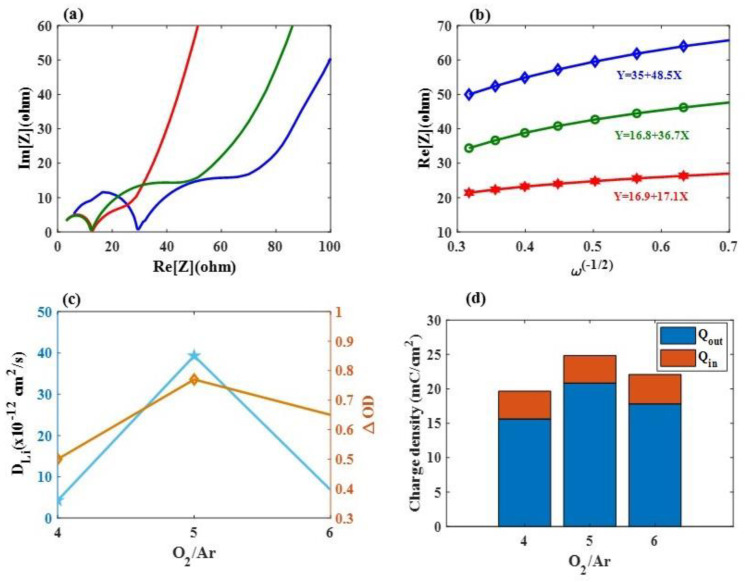
(**a**) Nyquist plots of various Ar/O_2_ gas flow ratios (Sample 1–Sample 3) from 100 kHz to 0.1 Hz, respectively; (**b**) the relationship between Z**’** and ω-1/2 for Samples 1, 2, and 3 under a low-frequency region; (**c**) the corresponding D_Li_ values and optical density (∆OD) at a wavelength of 633 nm for Samples 1, 2, and 3, respectively, and (**d**) R representing the rate of ion reversibility for Samples 1, 2 and 3.

Figure 2d illustrates the in situ optical transmittance of these Mo:WO_3_ films at a wavelength of 633 nm under the same voltage range from *−*1.5 V to 1 V, respectively, with different Ar/O_2_ gas flow ratios (1/4, 1/5, and 1/6, respectively) demonstrating the coloring and bleaching effects. The coloration and bleaching times were defined as the duration required for a 90% alteration in the full transmittance modulation. The coloration switching times (t_c_) and bleaching switching times (t_b_) were crucial factors for the system of Sample 2 calculated with a t_c_ of 9 s and a t_b_ of 9.8 s, respectively. In general, electrochromic materials containing active properties exhibit a relatively slow response time due to their low electron transport conductivity. Electrochemical impedance spectroscopy (EIS) tools are therefore used to conduct measurements on three-electrode systems, including for this type of cell being assessed. This cell consists of a working electrode (a Mo:WO_3_ film on ITO/glass), a counter electrode (Pt mesh), and a reference electrode (Ag/AgCl) in a 0.5 M LiClO_4_/Perchlorate (LiClO_4_/PC) solution. Figure 3a presents the corresponding Nyquist plots and analyzes the comparison of the charge transport kinetics between these Mo-doped WO_3_/ITO/glass films under various Ar/O_2_ gas flow ratios (Sample 1–Sample 3). Each sample contains two distinct parts: a semicircle at high and medium frequencies, and a straight line at low frequencies. The semicircle at high frequencies represents the resistance to Li+ ion migration across the electrode–electrolyte interface (R_f_), while the semicircle at medium frequencies reflects the charge transfer reaction (R_ct_) [6]; the oblique line represents Li^+^ ion diffusion to the electrodes, which is associated with the Warburg impedance [7]. As shown in Figure 2b, the point where the Re [Z] (ohm) axis intersects at the point of high frequency indicates the solution resistance (R_s_) [3]. The values of R_s_ for Sample 1 (0.02 Ω) were similar to those of Sample 2 (0.03 Ω) and Sample 3 (0.01 Ω). Samples 1, 2, and 3 exhibited charge transfer resistances (R_ct_) of 35 Ω, 16.9 Ω, and 17.1 Ω, respectively. The R_ct_ value obtained for Sample 2 was the lowest compared to the Mo:WO_3_/ITO/glass, which may be due to Sample 2 having a larger enclosed area of the CV curves. This leads to faster charge transfer at the electrode–electrolyte interface [35], which can significantly contribute to larger diffusion coefficients of the electrons and Li^+^ ions around the surface of the Mo:WO_3_/ITO/glass, thus enhancing the electrochemical response as a result [38,49].

In addition, Equation (2) can be used to calculate the diffusion coefficient D_Li_ (unit: cm^2^ s*^−^*^1^) of Li^+^ ions during the injection as well as the extraction of these ions into and out of the Mo:WO_3_/ITO/glass:

D_Li_ = R^2^T^2^/(2A^2^n^4^F^4^C_0_^2^σ^2^), (2)

where R is the gas constant, T is the absolute temperature of the experiment, A is the surface area of the electrodes, n is the number of electrons per molecule during oxidation, F is the Faraday constant, and C_0_ is the concentration of the Li^+^ ion in the Mo:WO_3_ electrodes [42]. The Warburg factor is calculated using the slope of Equation (2):

Z’ = R_s_+ R_ct_ + σω*^−^*^1/2^, (3)

where ω stands for the angular frequency. As shown in Figure 3b, σ (Ω cm^2^/s *^−^*^1/2^) values for the cathodic coloring electrodes were calculated based on the linear correlation between Z*’* and ω*^−^*^1/2^; Samples 1, 2, and 3 exhibited σ values of 48.5 Ω cm^2^/s *^−^*^1/2^, 17.1 Ω cm^2^/s *^−^*^1/2^, and 36.7 Ω cm^2^/s *^−^*^1/2^, respectively. The corresponding D_Li_ values were calculated using Equation (3) as displayed in Figure 3c). Sample 2 showed a significantly higher value (3.93 *×* 10*^−^*^11^ cm^2^ s*^−^*^1^) compared to Sample 1 (4.28 *×* 10*^−^*^12^ cm^2^ s*^−^*^1^) and Sample 3 (6.81 *×* 10*^−^*^12^ cm^2^ s*^−^*^1^). This can be attributed to the presence of nanostructures, which provide more channels for the movement of lithium ions and electrons [32]. Sample 2 also showed a significantly higher value of optical density (∆OD), which was defined as ln (T_bleaching_/T_coloration_) at a wavelength of 633 nm (0.77), than Sample 1 (0.5) and Sample 3 (0.65). Sample 2 can rapidly supply electrons to the surface of the Mo:WO_3_ layers, resulting in an enhanced ambipolar (ionic and electronic) diffusion into and out of these EC electrodes [42].

Based on these findings, it can be concluded that Samples 1*–*3 demonstrate an enhanced Li^+^ ion diffusion rate along with a reduced charge transfer resistance, leading to improved electrode kinetics for the Mo:WO_3_/ITO/glass-based coloration/bleaching process, resulting in a significantly improved performance [30]. R is defined as Q_out_/Q_in_, representing ion reversibility, and Samples 1, 2 and 3 exhibited R values of 79.54%, 83.93%, and 80.1%, respectively (see Figure 3d).

**Figure 4:**
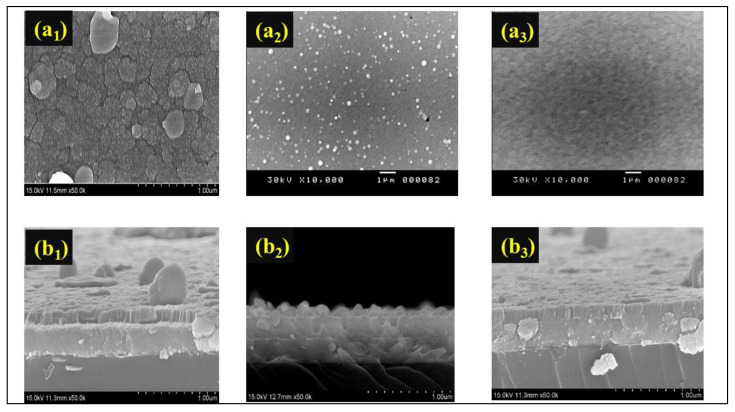
(**a_1_**–**a_3_**) top-view SEM images of the Mo:WO_3_ films for Sample 1–Sample 3; and (**b_1_**–**b_3_**) cross-sectional morphology images of the Mo:WO_3_ films for Sample 1–Sample 3.

Figure 4 displays the top-view SEM (Figure 4(a_1_–a_3_)) and cross-sectional morphologies (Figure 4(b_1_–b_3_)) of the Mo:WO_3_ film images of Samples 1, 2, and 3 indicating a consistent thickness of 200 nm. The particle size is inversely proportional to the flow of increasing O_2_ gas. To reduce the spot residence time, high-speed steering of the arc spot was employed across the surface of the cathodic target. By altering the O_2_ gas, the surface of these Mo:WO_3_ films may be polished to the point where the particles are reduced to macro-particles (MPs). The SEM image of Sample 2 shows the formation of nano-grains with a close-packed structure, resulting in nanoporous structures. In the SEM study of the surface morphologies along with the possible morphological changes of O_2_ gas, in general, the as-prepared Mo:WO_3_ films have a featureless surface morphology. Figure 4(a_1_) shows the rough but not uneven surface of the low value for O_2_ gas mass flow, which comprises dozens of nanometer-sized nanoparticles. Small Mo:WO_3_ film colloidal particles were formed by the hydrolysis of polycotton, which also led to cracks on the surface of the Mo:WO_3_ films. These nanoparticles provide efficient contact with the electrolyte solution. However, Figure 4(b_3_) displays an increasing O_2_ gas content, with the grain boundary having become blurry and disappeared as a result. The high O_2_ gas mass flow of Mo:WO_3_ became smoother and low compact compared to the low O_2_ gas Mo:WO_3_ films, thereby decreasing their surface area. Li diffusion within the material was slower and more difficult compared to that observed in a liquid electrolyte or along the grain boundary. This factor may explain the smaller charge capacities of the appropriate Mo:WO_3_ films that were observed for Sample 2 during the initial charge/discharge cycles.

### 3.2. Characteristics o thef Ir:NiO/ITO Films

**Figure 5:**
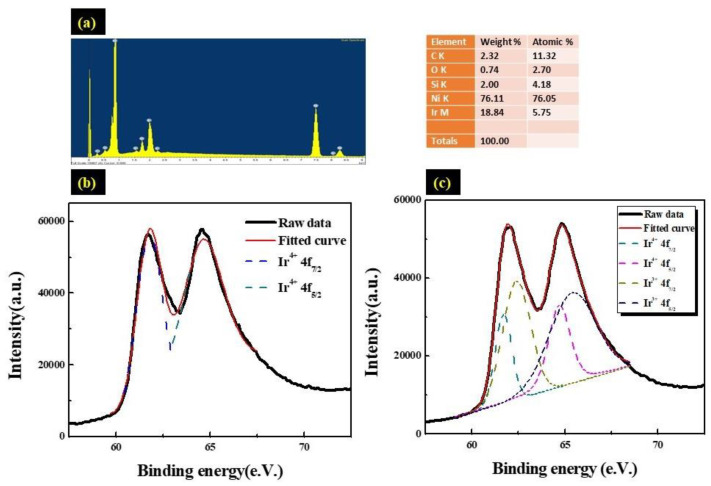
(**a**) EDS spectrum of the Ir:NiO/ITO glass; (**b**) high-resolution XPS Ir4f spectra displaying the effects of bleaching the Ir:NiO/ITO flim at a 100 nm thickness; and (**c**) high-resolution XPS Ir4f spectra displaying the effects of coloring the Ir:NiO/ITO flim at a 100 nm thickness.

The chemical composition of the Ir:NiO/ ITO film was assessed using the EDS spectrum, as shown in Figure 5a. The elemental composition of the Ir:NiO film, including the atomic percentage (at%) and weight percentage (wt%), is displayed in the inset. The table shows the presence of iridium (Ir), nickel (Ni), and oxygen (O).

The elements Ir and Ni are present in Ir:NiO; Sn and In are present in the ITO substrate, while O is present in both Ir:NiO and the ITO substrate. The absence of peaks other than those observed of the ITO substrate were determined to be attributable to the Ir, Ni and O elements, and confirmed the deposition of an Ir:NiO film without any elemental impurities. The surface composition of the Ir:NiO/ ITO films prepared with the CAP technique was analyzed using X-ray photoemission spectrum (XPS). Electrochemical testing of the IrO_2_-doped Li_x_ (NiO) was performed in a 0.5 M liquid–electrolyte solution of LiClO_4_/ PC using a three-electrode cell. The cell consisted of a working electrode (Ir:NiO film on ITO/glass), a counter electrode (Pt mesh), and a reference electrode (Ag/AgCl). Figure 5c shows that the peaks Ir 4f_7/2_ and 4f_5/2_ are located at the binding energies of 61.7 eV and 64.7 eV, corresponding to the Ir4 f_5/2_ and Ir 4 f_7/2_ peaks of the Ir^3+^ ion in Li_x_IrO_2_, respectively. The coloration process indicates the movement of the Li^+^ ions and electrons into the Ir:NiO/ITO films, such that the Ir^3+^ extracted an e^−^ to become Ir^4+^, resulting in a corresponding shift in the peak to a lower energy level. As shown in Figure 5c, the evaluated ions transformed from the Ir^3+^ to the Ir^4+^ state, and it was calculated that approximately 35% (100 nm) of the ions transformed from the Ir^3+^ to the Ir^4+^ state. Thus, we can deduce that only Ir^3+^ ions were present in the 100nm-thick Ir:NiO/ITO films under the bleached state (as shown in Figure 5b).

### 3.3. Bi-Functional ESECDs: Electrochromic and Energy-Storge Performance

**Figure 6:**
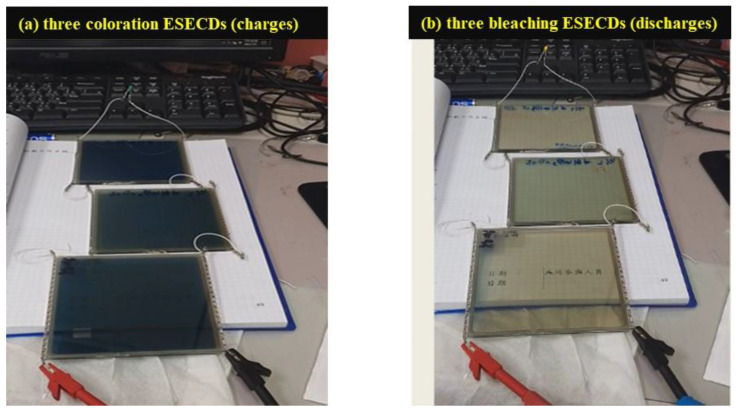
(**a**) Digital image of the connection of three ESECDs under the coloration state; and (**b**) three ESECDs under the bleaching state (discharge) were able to light the yellow LED, which contributes a bi-functional electrochromic supercapacitor.

To demonstrate the potential of a working layer that utilizes Mo:WO_3_ films (with an O_2_/Ar ratio of 5 at the thickness of 200 nm) and a counter layer consisting of Ir:NiO films (with an O_2_/Ar ratio of 3 at the thickness of 100 nm), a bi-functional electrochromic supercapacitor device was constructed. Figure 6 displays a digital photograph of the ESECDs (glass/ITO/Mo:WO_3_/gel polymer electrolyte/Ir:NiO films/ITO/glass) assessed under both the coloration and bleaching states. The active area of the ESECDs was 10 × 10 cm^2^. The optical images of these ESECDs revealed a deep blue coloration state under a negative potential of *−*2.2 V. Once a reverse potential of +2 V was applied, the ESECDs showed a bleaching state. Furthermore, Figure 6 reveals that a series connection of three colored states (charges) based on Ir:NiO ESECDs were able to illuminate a 2.15 V yellow LED, indicating the practical applicability of these devices as energy storage systems for EC smart windows. The Ir:NiO ESECDs demonstrate an integrated energy storage, and the color variations resulting from this energy can be used to indicate the charge–discharge state of the device.

In general, ESECDs with a high durability and stability are required to prevent the accumulation of trapped ions (*Q_trap_*). The accumulated *Q_trap_* can be calculated as follows [37,38]:

(4) $$Q_{trap}=\int_{1}^{m}\left\{\left(1-R\right)\times Q_{in}\right\}dn$$

where *Q_in_* represents the amount of inserted ions, and *R* represents ion reversibility, which is expressed as the ratio of ions extracted to ions inserted. The value of *Q_trap_* depends on the *Q_in_* and *R*. In addition, the charge density of the inserted ions and extracted ions can be calculated by the integration of the CV curves [39],

(5) $$Q_{in}=\int^{\;}IdV/v$$

which complies the following Equation (5).

**Figure 7:**
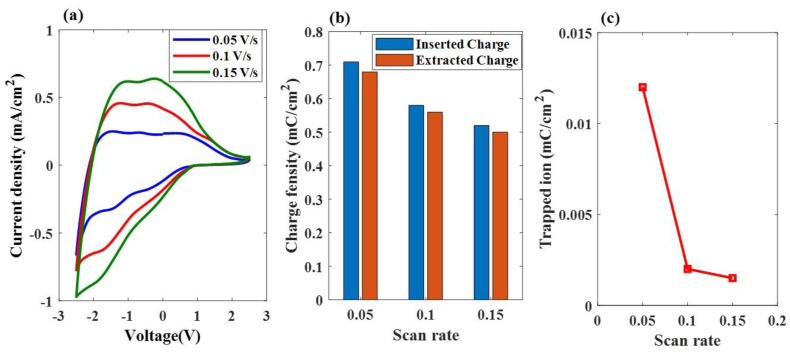
Electrochemical properties and electrochromic performances of the ESECDs under different scan rates via the CV test. (**a**) CV curves. (**b**) The charge density of the inserted ions and extracted ions. (**c**) The relationship between the trapped ions and the scan rates.

Figure 7a shows the plot of the current density versus voltage during the first cycle of the applied sweep voltage, ranging from *−*2.5 V to 2.5 V, at three scanning rates of 0.05 V/s, 0.10 V/s, and 0.15 V/s, respectively. Based on the CV curve, it can be observed that the area under the curve for all three types of ESECDs increased as the scan rate also increased. The charge densities of the inserted ions, calculated by Equation (5), are shown in Figure 7b. Based on Equations (4) and (5), the charge density varied directly with the area under the CV curve, but inversely with the scan rate. ESECDs exhibited the lowest Q_trap_ under the 0.15 V/s scanning rate, which was smaller than that observed of 0.1 V/s and 0.05 V/s. Figure 7c shows the relationship between the charge of the trapped ions and the scan rates, demonstrating that slower scan rates lead to the formation of more trapped ions in these ESECDs.

**Figure 8:**
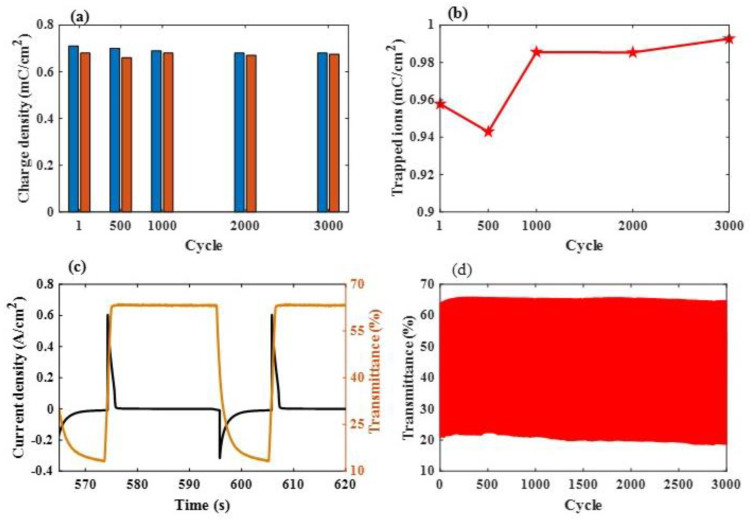
(**a**) Evolution of the charge density of the inserted ions in the ESECDs under different scanning rates with 0.15 V/s; and (**b**) the evolution of the reversibility between the inserted ions and the extracted ions; (**c**) chronoamperometry (CA) response time with in situ transmittance measurements of the Ir:NiO ESECDs under their coloration and bleaching states for 10 s; and (**d**) the durability of the Ir:NiO ESECDs (glass/ITO/Mo:WO_3_/gel polymer electrolytes/Ir:NiO/ITO/glass) up to 3000 times.

Figure 8a presents the durability of the ESECDs that have undergone up to a 1000 CV cycles under a sweep voltage range from −2.5 V to 2.5 V, respectively, and a scanning rate of 0.15 V. As shown in Figure 8b, the reversibility (R for ESECDs) of the Ir:NiO -based ESECDs was around from 0.96 to 0.95, respectively. It seems that the high R value reduces the amount of trapped ions and the probability of ion blockage, which affects the driving force and the time of the ion insertion process. Figure 8c presents the electrochromic performance of the Ir:NiO ESECDs (glass/ITO/Mo:WO_3_/gel polymer electrolytes/ Ir:NiO/ITO/glass) with an active area of 10 × 10 cm^2^. Figure 8c also presents the in situ transmittance of these ESECDs, as analyzed during a continuous potential cycle from −2.2 V (coloration potential, V_c_) to 2 V (bleaching potential, V_b_), respectively. Figure 8c shows that the coloration (charge process) and bleaching states (discharge process) of the ESECDs were measured via CA curves and in situ optical responses of transmittance at a fixed 633 nm. The coloration and bleaching of these switching times or speed was a prominent characteristic of the ESECD system, which was defined as the time required for a 90% change in the full transmittance modulation.

As shown in Figure 8c, a maximum optical modulation was reached at 53% (from T_bleaching_ (66.6%) to T_coloration_ (13.1%)) and the switching times were obtained at a wavelength of 633 nm. Figure 8c illustrates the ESECD switching times at a wavelength of 633 nm coloration time of 3.58 s, and a rapid bleaching time of 1.24 s. Notably, the optical transmittance modulation measured with the CA curves had the same trend as the optical transmittance modulation measured with the CV curves. The durable stability of the Ir:NiO ESECDs is an important factor that can be to determine whether the mass produce can work their functions in real life. Figure 8d presents the long-time 3000 cycles. After 3000 cycles of the bleaching/coloration operation, this led to the retainment of 96% (4% decayed) of its initial state. The high contrast optical performance and the good durability of these ESECDs could be attributed to the inserted MoO_3_-doped WO_3_ (Mo:WO_3_) and IrO_2_-doped NiO films for the electrochromic electrode. New anodic discoloration materials may provide new insights into developing energy-storage prominent candidates for their use in smart windows. In addition, we also surveyed the comparison of recent research on electrodes under various conditions, as detailed in Table 3.

## 4. Conclusions

We have investigated the influence of MoO_3_-doped WO3 (Mo:WO_3_) films under various Ar/O_2_ gas flow ratios (1/4, 1/5, and 1/6, respectively). According to the results we obtained, in Sample 2, the σ value was calculated based on the linear correlation between Z’ and ω-1/2, in which σ exhibited 17.1, and their corresponding D_Li_ was significantly of higher value (3.93 × 10^−11^ cm^2^ s^−1^). This can be attributed to the presence of nanostructures, which provide more channels for the movement of the Li ions and electrons. In our study, we have fabricated counter electrodes (ion storage layers) using IrO_2_-doping NiO (Ir:NiO) films, which could present as alternatives to the traditionally active materials for the bi-functional electrochromic batteries.

The ESECDs used in this study were 10 × 10 cm^2^ in size and achieved an optical transmittance modulation of the Ir:NiO ESECDs (glass/ITO/Mo:WO_3_/gel polymer electrolytes/Ir:NiO/ITO/glass), with ΔT = 53.3% (from T_bleaching_ (66.6%) to T_coloration_ (13.1%)). Furthermore, Figure 6 demonstrates that a series connection of three colored states (charges) based on the Ir:NiO-ESECDs were able to illuminate a 2.15 V yellow LED, indicating their practical applicability as energy-storage systems for EC smart windows. We also present the durability of the ESECDs up to 3000 CV cycles and the reversibility (R for ESECDs) of the Ir:NiO-based ESECDs was determined to be from 0.96 to 0.95, respectively. After 3000 cycles of the bleaching/coloration operation, the ESECDs were able to retain 96% (4% decayed) of their initial state. The high contrast optical performance and good durability of these ESECDs could therefore be attributed to the inserted MoO_3_-doped WO_3_ (Mo:WO_3_) and IrO_2_-doped NiO films for the electrochromic electrode.

## Figures and Tables

**Table 1:** The deposition parameters for both the electrochromic layer and the transparent conducting layer was determined.

No.	Film	Ar/O_2_ (Ar = 150 sccm)	W.P. (Torr)	DC Power (W)	Deposition Temp. (°C)	Deposition Time (s)	Thickness (nm)
Sample 1	Mo:WO_3_	1/4	1 × 10^−2^	1400	50	1450	200
Sample 2	Mo:WO_3_	1/5	1.5 × 10^−2^	1400	50	1500	200
Sample 3	Mo:WO_3_	1/6	3 × 10^−2^	1400	50	1550	200
	ITO	Ar = 150	3.5 × 10^−3^	650	200	3600	300

**Table 2:** Deposition parameters of the WO_3_ electrode film and the ITO glass.

Target	Film	Ar/O_2_ (Ar = 150 sccm)	W.P. (Torr)	DC Power (W)	Deposition Time (s)	Deposition Rate (nm/s)	Deposition Temp °C	Thickness (nm)
Ir/Ni Metal	Ir: NiO	1/3	8 × 10^−3^	1500	100	1	50	100

**Table 3:** Comparison of the recent research on electrodes under various conditions.

Materials/Device	Method	∆T (%)	CE (cm^2^*/*C)	Switching Time (t_c_/t_b_)	Refs.
Ir:NiO/Mo:WO_3_	CAP	53%		3.58/1.24	This work
IrO_2_ buffer Ti:V_2_O_5_/WO_3_	CAP	57	96.1	4.0/1.4 s	[22]
IrO_2_/WO_3_	CAP	50	-	4.8/1.5 s	[10]
V_2_O_5_/WO_3_	Polyol-mediated synthesis	50	-	-	[49]
V_2_O_5_/WO_3_	Spin coating	31	-	8.2/6.3 s	[50]

## Data Availability

The data presented in this study are available on request from the corresponding author. The data are not publicly available due to privacy.
